# Aggression in Adolescents: The Role of Mother-Child Attachment and Self-Esteem

**DOI:** 10.3390/bs12050147

**Published:** 2022-05-17

**Authors:** Alif Muarifah, Riana Mashar, Intan Hashimah Mohd Hashim, Nurul Hidayati Rofiah, Fitriana Oktaviani

**Affiliations:** 1 Faculty of Teacher Training and Education, Ahmad Dahlan University, Yogyakarta 55166, Indonesia; riana.mashar@pgpaud.uad.ac.id; 2 School of Social Sciences, Universiti Sains Malaysia, Gelugor 11800, Penang, Malaysia; hashimah@usm.my; 3 Graduate School for International and Cooperation, Hiroshima University, Hiroshima 739-8511, Japan; d191200@hiroshima-u.ac.jp; 4 Faculty of Psychology, Ahmad Dahlan University, Yogyakarta 55166, Indonesia; fitriana1808044052@webmail.uad.ac.id

**Keywords:** adolescents, aggression, attachment, self-esteem, structural equation modeling

## Abstract

Every year, adolescents’ aggressive behavior in the world continues to increase, including in Yogyakarta, Indonesia. Teenagers’ aggressive behavior in Yogyakarta leads to criminal acts resulting in physical sacrifices and death. The aggressive behavior of teenagers is known as *Klithih*. It develops and continues to increase every year, causing public anxiety and concern. This study aimed to examine the role of mother–child attachment and self-esteem against aggression. Aggression, mother’s attachment, and self-esteem scales were deployed to collect data and were filled out by 730 high school students between 15 and 19 years old from twenty-three schools in five municipalities in Yogyakarta Province (M = 16.52, SD = 0.793, 310 male students and 420 female students). Descriptive statistics described research data by IBM SPSS 23 and structural equation modeling by AMOS v20 to test research hypotheses. The results of the study showed a good fit, indicating that self-esteem might enhance the effect of each mother’s attachment style on aggression. Our study showed that insecure attachment positively and significantly affected aggression and negatively and significantly affected self-esteem. Furthermore, it also revealed that anxious attachment positively and significantly influenced aggression and negatively and significantly influenced self-esteem. Lastly, our finding revealed that self-esteem negatively and significantly affected aggression. These findings suggested that better mother’s attachment and higher self-esteem in adolescents may lower the possibility of aggression, whereas insecure attachment, anxious attachment, and low self-esteem may increase the risk of aggression.

## 1. Introduction

Aggression is a primitive behavior passed down from generation to generation from time immemorial, is persistent enough that it is near impossible wipe out completely, is committed on purpose, and is directly and indirectly expressed through wrath [1]. Aggression is expressed by harsh behavior both physically and verbally, hence endangering human beings and other living beings and causing difficulties, damages to property, and pain. Harsh behavior of aggression is from the simplest to the most complex levels [2]. Aggression is a purposive act to hurt oneself, others, or the environment physically and verbally [3]. Verbal aggression is most frequently committed and experienced by teenagers. This behavior contributes to physical aggression, especially toward adolescents with emotional disorders [4]. Verbal aggression is directly and indirectly expressed through anger and feud, causing negative thinking, damaging integrity and social relationships, threatening security, and hurting the welfare of children, adolescents, and adults [1]. Acts of physical aggression include punching, kicking, shooting, or even killing [5].

Aggression is linked to potential mental health issues [6]. It is linked to signs of depression [7] and negative morals [8]. Weak self-control may lead to aggressive behavior [9]. Because it causes injuries physically and verbally, it is better to avoid being the target of aggression [10]. Experiences of falling victim to physical aggression are highly likely to inspire aggressive behavior in the future [11]. The resulting consequences are negative thinking, damaged integrity and social relationships, disturbance to security, death, and mental disorders, such as failure in adapting, despair, depression, suicide, and alcoholism [4,5,12,13,14,15]. It is a protective response to pressure and distress along with a sense of anger that is expressed by hurting others, intimidating, or asserting dominance [16]. Most aggressive individuals fail at adapting both in their family and at school [12]. Aggression that is directed toward peers may cause physical wounds, psychosocial and academic problems, and strenuous relationships with peers [15]. Verbal aggression includes insults that cause emotional and psychological problems as they relate to self-competence [17,18]. The victim of aggression often develops suicidal thoughts due to the pressure that they are facing [19].

Aggressive behavior is on the front line of the causes of global adolescent morbidity and mortality. As much as 5.5% mortality in adolescents is caused by aggressive behavior. Research in the U.S. showed that 22.6% of adolescents have engaged in physical fights [20]. A study in Spain found an average score for verbal aggression of 57.60% [21]. Individuals with low levels of self-control are highly likely to show aggressive behavior [22]. In China, 39.2% experienced medium levels of physical aggression [23]. Meanwhile, among Jakarta teenagers, 85.7% perpetrated aggression in the score range 68–106 [24]. In Russia, up to 80% of adolescents committed verbal aggression [25]. In India, 46.04% experienced physical aggression [26]. Seventy-three percent of male *adolescents* in India showed moderate levels of aggressive behavior and thirty-two percent did high levels of aggressive behavior. It was further discovered that 57% of female adolescents had moderate levels of aggressive behavior and 18.5% did high levels of aggressive behavior [27]. In Iran, it was found that the aggressive behavior in adolescents averaged a score of 63.61% [28].

Several studies related to aggression have been documented in several countries where the results are in line with the results of research in Indonesia [29,30,31]. Research in Indonesia with 2681 adolescent subjects showed that adolescents committed physical and verbal aggression in many places, although the levels of aggression in rural areas were not as high as they were in other locations [31]. Other research in Indonesia showed that 11.9% of the adolescents demonstrated high levels of aggressive behavior and 75% did moderate levels of aggressive behavior [29]. A study in Banda Aceh found that 81.48% of the teenagers showed aggressive behavior [30].

The latest data explains that aggressive behavior in the Special Region of Yogyakarta, called *Klithih*, carried out by teenagers on the streets has caused child casualties from members of the Regional People’s Representative Council [32]. *Klithih* is an act of violence on the streets in the form of fights, throwing stones, injuring, and even killing the victim, directed to other people at random [33]. This requires special attention to find the right solution considering that there has been an increase in sexual behavior by adolescents [34]. In 2022 the local police arrested 99 juvenile offenders (14–19 years old) [35]. Previous studies have explained that family resilience contributes to the prevention of *Klithih* [36], which explains the vital role of the family. Besides, in Indonesia, the mother acts as the primary caregiver compared with the father, who serves as the breadwinner [37]. Therefore, the focus of this research was on mother–child attachment. In addition, personal factors are important to study so that adolescents have stable self-esteem and prudence in acting [38].

## 2. Literature Review

### 2.1. Attachment and Aggression

It is essential to examine adolescent aggression and the factors associated with aggression. Previous studies found that family factors, such as insecure attachment and anxious attachment, are closely associated with aggressive behavior [39,40,41]. Attachment is a quality comprising a mutual emotional bond between the infant and the caregiver that influences the infant’s character development [42]. Attachment is divided into secure attachment and insecure attachment (anxious attachment and avoidant attachment) [42]. Secure attachment between the child and the caregiver creates emotional stability, allows for good self-control ability development, encourages social interaction, develops self-esteem, confers strength against pressure, and prevents mental disorders and aggressive behavior [43]. Secure attachment as a learning experience has a role in low aggressiveness [44]. Primary affection in parenting creates emotional stability and a sense of safety, hence producing low levels of aggression [45].

Children with anxious attachment experience non-responsive and inconsistent treatment in fulfillment of their needs [42]. Those children learn not to express themselves and their needs, leading to a poor attachment, which eventually results in fear of others’ rejection [42]. Children will have an insecure attachment (avoidant attachment) when they experience a treatment that consistently does not fulfill their needs [42]. The attachment quality of an individual with the parents tends to contribute significantly to the development of aggressive behavior [46]. Dysfunctional anger and aggression are the core of insecure attachment [46,47]. Children experience conflict between their need for a close relationship and their wishes regarding other people’s responses [48].

Previous studies showed that anxious attachment affects physical aggression and hostility more greatly than other types of insecure attachment [39]. A study on 388 high school students with a regression analysis technique found that attachment had an effective contribution to aggression of 16.5%. Insecure attachment had a significant effect on aggressive behavior in the form of physical aggression, verbal aggression, or hostility [39]. Learning experience with emotional attachments may reduce depression, stress, and other negative emotions [49,50]. Insecure attachment causes children to behave negatively in expressing their emotions. They somehow become reclusive individuals; they cannot depend on others, and their self-control against impulses is low [51]. All types of insecure attachment have a positive relationship with both physical and verbal aggression [52]. Insecure attachment significantly affects a child’s ability to express his/her emotions, hence triggering aggressive behavior [41].

Therefore, the stronger the secure attachment with the parents, the more likely the child avoids aggressive behavior [40]. Insecure attachment may strengthen the proactive aggression predisposition in males. The lower the secure attachment, the higher the aggressive behavior reactivity [53]. Contrarily, strong secure attachment helps a child adapt to the environment, create harmonious relationships, and control their emotions [54]. Attachment contributes crucially to future behavior prediction [55]. Providing prime affection may create a child’s emotional stability since it triggers a sense of security and comfort [45]. Individuals with insecure attachment have difficulties in controlling emotions and in trusting others and get anxious easily [56]. Other research results discovered that secure attachment is linked to low levels of aggressive behavior in children and to low levels of adolescent engagement in other dangerous behaviors [43].

### 2.2. Attachment, Self-Esteem, and Aggression

Other research also found that the quality of child-parent attachment could influence self-esteem [57] and later significantly influence aggressiveness [58]. Some findings proved that secure attachment and insecure attachment as fundamental learning experiences have a significant effect on an individual’s ability to conduct self-evaluation and control positive and negative behaviors, thereby being useful for both the individual and their environment [59].

There are two factors at play in the development of self-esteem: reflected appraisals and social comparisons. It is presumed that there is a susceptibility to depression, drug use, and violence [60]. Individuals who manage to develop their self-esteem can defend themselves, be loved and accepted by others, show warm, responsive behavior, and have high self-efficacy [61,62]. Individuals with high self-esteem have respect for values, their morals are competent, and they exhibit achievement in academic fields [63]. They have a positive attitude toward themselves and others [64], good emotional regulation, and perseverance [65]. Other studies found that adolescent aggression and antisocial behavior are expressions of low self-esteem; this is formed in the learning process with their parents [66]. Secure attachment is the most crucial determinant for stable self-esteem [67]. Individuals with high self-esteem are usually less aggressive, while individuals with low self-esteem show otherwise [68].

Some studies also showed that parenting and self-esteem are correlated with aggression [69]. Self-esteem is formed through a long process [70]. Individuals with low self-esteem will protect themselves from a sense of being rejected, inferiority, and shame for failures, hence tending to show aggressiveness and violence toward others. Individuals with high self-esteem do not easily become stressed, being more confident in facing the environment’s demand; hence, they can prevent pathological symptoms such as aggression [71]. Aggressiveness that is related to negative emotions, reactive aggressiveness, and adaptive functions reflect criminal lifestyles and problematic, irresponsible, impulsive behaviors [72]. Adolescents with secure attachment tend to have higher self-esteem than those with insecure attachment (ambivalent). Self-esteem is negatively related to aggressive behavior [73], while insecure attachment and anxious attachment are related to unstable self-esteem [74]. The quality of secure attachment influences the development of self-esteem and significantly influences an individual in controlling their behavior [46].

Self-esteem and aggression are significantly correlated with each other [58]. Individuals with low self-esteem are prone to greater reactive aggression because of anger and hostility [75]. Whether it be a positive or negative treatment, how a mother treats her child affects her attachment with the child, thus affecting the child’s self-esteem and behavior [57]. Other research also discovered that low self-esteem could increase peers’ rejection and trigger aggressive acts in adolescents at the high school level [75]. Self-esteem has a negative effect in women with or without criminal records [76]. Self-esteem was also found to mediate or to be negatively related to isolation and aggression in high school adolescents [77]. The warmth built with parents will inspire self-image about one’s self and others that can be used as a guide to control behavior [59].

### 2.3. Present Study

The primary aim of the present study was to examine the mediating role of self-esteem in the effect of mother’s attachment styles on aggression among adolescents in Yogyakarta, Indonesia. Based on previous research (e.g., Gomez and McLaren [78]), we hypothesized that self-esteem would moderate the effect of mother’s attachment styles (i.e., secure attachment, insecure attachment (avoidant attachment), and anxious attachment) on aggression. So far, research on self-esteem as a mediation of the relationship between mother–child attachment and aggression has not been studied in Indonesia. More specifically, each mother’s attachment style has a different effect on aggression and self-esteem. We believed that our study would provide valuable information regarding the role of mother–child attachment and self-esteem in adolescents’ aggression.

## 3. Materials and Methods

### 3.1. Participants and Procedure

A total of 730 high school students aged 15–19 years old (M = 16.52, SD = 0.793), comprising 310 male students and 420 female students, participated in the present study. The participants were selected from twenty-three schools in five municipalities in Yogyakarta Province (i.e., Bantul, Kulonprogo, Sleman, Wonosari, and Yogyakarta). This study obtained a research permit from Ahmad Dahlan University, the Yogyakarta provincial government, the Department of Education, and schools.

We asked school counselors to identify students for participation in our research. The participants were selected based on several criteria: grade XI students, living with parents, and having a mother who was at least a high school graduate, having a job, and having a maximum of four biological children. Once the participants were selected, the researchers set the schedule for data collection. The researchers visited the schools and distributed the instruments to the students. In completing the instruments, the participants were assisted by six research assistants and twenty-three school counselors. Informed consent was obtained from the participants. We also explained the project’s purpose and procedure. The participants were informed that their data and responses would be kept confidential. The data collection phase was carried out for two months. Each participant received a voucher valued IDR 50,000.00 for their participation.

### 3.2. Measures

#### 3.2.1. Aggression

Adolescents’ aggression was measured by a 33-item Likert scale (α = 0.915), designed based on the theory proposed by Baron and Neuman [79]. The items covered eight forms of aggression, namely (A) direct active physical, (B) direct passive physical, (C) indirect active physical, (D) indirect passive physical, (E) direct active verbal, (F) direct passive verbal, (G) indirect active verbal, and (H) indirect passive verbal. Some of the items included, e.g., “I will kick people who often make trouble” and “I will spread gossip about friends who often hurt others”. The seven-point Likert scale ranged from 1 (very low intensity) to 7 (very high intensity). The total score reflected the intensity of aggression.

#### 3.2.2. Mother’s Attachment

Mother’s attachment was assessed using 31 items designed based on the theory proposed by Ainsworth [80]. The scale consisted of three sub-scales to evaluate the intensity of mother–child attachment. The three sub-scales were (A) secure attachment (α = 0.957), e.g., “The mother was a faithful and communicative listener”, (B) insecure attachment (α = 0.726), e.g., “The mother never believed in the reason given by her child”, and (C) anxious attachment (α = 0.786), e.g., “The mother leaves her child in loneliness when facing problems”. This scale was a seven-point Likert scale ranging from 1 (very low intensity) to 7 (very high intensity).

#### 3.2.3. Self-Esteem

We assessed adolescents’ self-esteem using a 35-item Likert scale (α = 0.953), designed based on the theory by Baumesiter [81], Coopersmith [61], and Dutton and Brown [82]. The indicators of self-esteem in this study were (a) self-management, (b) responsiveness and adaptiveness, (c) self-esteem, (d) having competence and achievement, (e) having self-capacity, and (f) value adherence. The seven-point Likert scale ranged from 1 (very low intensity) to 7 (very high intensity). Some of the items included, e.g., “I dare to compete with friends”, “I accept offers according to ability”, and “I admit and apologize for breaking the rules”. The total score reflected the self-esteem level; low intensity indicated low self-esteem, while high intensity indicated high self-esteem.

### 3.3. Data Analysis

In this study, the data were analyzed using descriptive statistics by IBM SPSS 23 to describe the research data and structural equation modeling (SEM) with AMOS v20 Software to examine the validity of theoretical models and the relationship between variables. The strength of the variable correlation coefficient refers to Dancey and Reidy [83], which can be seen in Table 1. The model fit was assessed based on Goodness of Fit Index (GFI), where GFI = 1.00 indicated perfect fit and GFI > 0.9 indicated a good fit, Adjusted GFI (AGFI), where AGFI close to 1.00 indicated a good fit, Root Mean Square Error of Approximation (RMSEA), where RMSEA < 0.05 indicated a good fit, Normed Fit Index (NFI) (NFI ≥ 0.90), Comparative Fit Index (CFI) (CFI ≥ 0.90), and Tucker–Lewis Index (TLI) (TLI > 0.90) [84]. A study should report at least three fit indices to gain a good or perfect fit model [85].

## 4. Results

### 4.1. Descriptive Statistics

**Figure 1:**
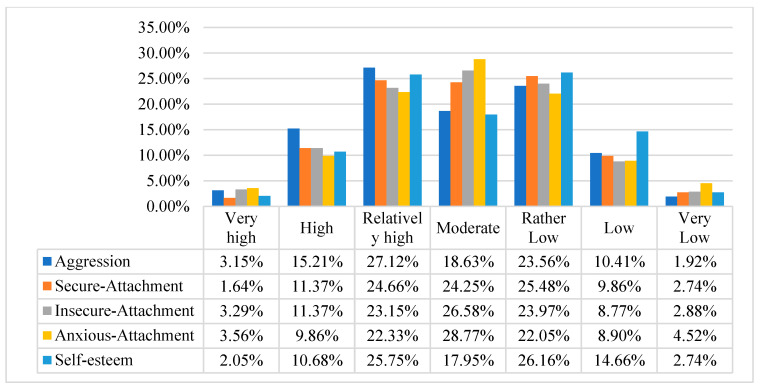
The distribution of scores of the variables.

The mean, standard deviation, minimum score, and maximum score are shown in Table 2. The distribution of the variables’ scores is reported in Figure 1.

This study found that most adolescents had a relatively high intensity of aggression (27.12%), rather low intensity of secure attachment (25.48%) and self-esteem (26.16%), and moderate intensity of anxious attachment (28.77%) and insecure attachment (26.58%). However, the variable scores were found to be slightly different in three categories (i.e., relatively high, somewhat low, and moderate).

The correlation between variables is shown in Table 3. Secure attachment was found to be significantly and negatively correlated with aggression (r = −0.216, *p* < 0.001) and positively and significantly correlated with self-esteem (r = 0.421, *p* < 0.001). In contrast, insecure attachment was found to be significantly and positively correlated with aggression (r = 0.141, *p* < 0.001) and negatively and significantly correlated with self-esteem (r = −0.280, *p* < 0.001). Anxious attachment was positively correlated with aggression (r = 0.172, *p* < 0.001) and significantly and negatively correlated with self-esteem (r = −340, *p* < 0.001). It was also found that self-esteem exhibited a significant, negative correlation with aggression (r = −0.402, *p* < 0.001). Referring to Dancey and Reidy [83], the results of correlation analysis showed that each type of attachment has a weak correlation to aggression, and self-esteem has a moderate correlation to aggression. Secure attachment has a moderate correlation to self-esteem, while insecure attachment and anxious attachment have a weak correlation to self-esteem.

### 4.2. Hypothesis Analysis

The role of secure attachment, insecure attachment, anxious attachment, and self-esteem in aggression were analyzed. The data analysis results, as shown in Table 4, indicated different significant effects between variables. First, the results indicated that secure attachment had a negative effect on aggression (β = −0.225, *p* < 0.001) and a positive effect on self-esteem (β = 0.402, *p* < 0.001). The results also revealed that insecure attachment had a positive effect on aggression (β = −0.147, *p* < 0.001) and a negative effect on self-esteem (β = 0.267, *p* < 0.001). Moreover, this study found that anxious attachment had a positive effect on aggression (β = −0.174, *p* < 0.001) and a negative effect on self-esteem (β = 0.314, *p* < 0.001). Another finding showed that self-esteem had a negative effect on aggression (R2 = −0.441, *p* < 0.001).

Then, we conducted a one-way analysis to see the role of self-esteem as a mediator since it had fulfilled three conditions of mediator testing, namely (1) the independent variable had a significant effect on the hypothesized mediator; (2) the independent variable had a significant effect on the dependent variable; and (3) the mediator had a significant effect on the dependent variable [85]. The analysis results showed that self-esteem as a mediator contributes to the relationship between each type of attachment and aggression. This is indicated by the difference in the number of direct effects and total effects.

**Figure 2:**
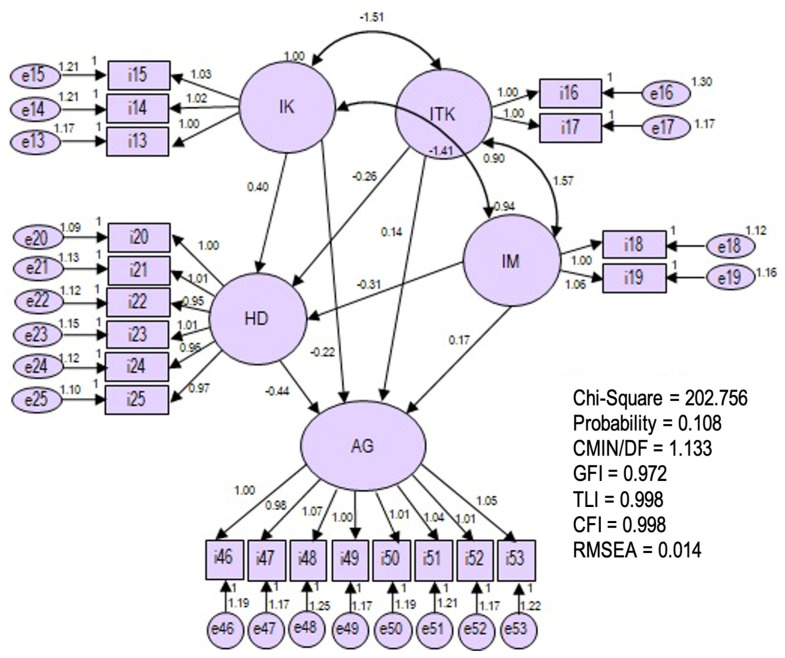
The final model paths. Note: Secure attachment (IK), Insecure Attachment (ITK), Anxious Attachment (IM), Self-Esteem (HD), Aggression (AG). The error standard (e13–e25; e46–e53). The indicator of secure attachment: warm (i13), responsive (i14), sensitive (i15). The indicator of insecure attachment: sceptical (i16), submissive (i17). The indicator of anxious attachment: insensitive (i18), less involved (i19). The indicator of self-esteem: self-regulation (i20), adaptive responsiveness (i21), self-assessment (i22), competence and achievement (i23), understanding self-capacity (i24), adherence to values (i25). Aggression indicators: direct physical active (i46), direct physical passive (i47), indirect physical active (i48), indirect physical passive (i49), direct verbal active (i50), direct verbal passive (i51), indirect verbal active (i52), passive indirect verbal (i53). The Minimum Sample Discrepancy Function Divided with degree of Freedom (CMIN/DF), Goodness of Fit Index (GFI), Tucker–Lewis Index (TLI), Comparative Fit Index (CFI), Root Mean Square Error of Approximation (RMSEA).

Figure 2 displays the final path model, and the hypothesized model showed a good fit model, which was proven by the Goodness of Fit Index (GFI) = 0.972, Tucker Lewis Index (TLI) = 0.998, Comparative Fit Index (CFI) = 0.998, and Root Mean Square Error of Approximation (RMSEA) = 0.014 [85].

The analysis results showed that self-esteem, as a mediator, significantly increased the effect of each attachment style on aggression. The indirect effect of self-esteem on secure attachment was valued as −0.177 (*p* < 0.001), and the total effect was −0.402 (*p* < 0.001). Then, the indirect effect of self-esteem on insecure attachment was valued as 0.118 (*p* < 0.001), and the total effect was 0.265, *p* < 0.001. Lastly, the indirect effect of self-esteem on anxious attachment was valued as 0.138 (*p* < 0.001), and the total effect was 0.312 (*p* < 0.001).

## 5. Discussion

The study results showed that each mother’s attachment style had a significant correlation with and effect on aggression. These findings suggested that a better attachment between the mother and the child could reduce the possibility of aggression in adolescents.

The study found that secure attachment negatively affected aggression, while insecure attachment and anxious attachment positively affected aggression. The study also showed that secure attachment had a greater significant effect on aggression than insecure and anxious attachment. These findings suggested that the mother’s secure attachment could result in an adolescent’s lower aggression. At the same time, insecure and anxious attachment would increase the risk of aggression. Previous studies by Brodie et al. also showed that insecure attachment negatively affected aggression [39]. This study explains that high school students with insecure attachments tend to do physical, verbal, and hostile aggression.

Another finding of the study showed that secure attachment positively affected self-esteem, while insecure attachment and anxious attachment did negatively. These findings suggested that better mother’s secure attachment could lead to higher self-esteem in adolescents. In contrast, insecure attachment and anxious attachment could lower adolescents’ self-esteem. Adolescents who had secure attachment with their parents reported better self-esteem than those with insecure attachment [86,87]. Another researcher also found that insecure attachment was negatively associated with self-esteem; this condition was caused by insecure and restless early parenting [88].

Our subsequent finding exhibited that self-esteem negatively affected aggression [58]. Our results indicated that high self-esteem could lead to lower aggression, and low self-esteem could increase aggression. These findings support previous studies, which found that individuals with high self-esteem could respect themselves and others and admit and accept their mistakes [89].

This study found that self-esteem, as a mediating variable, could enhance the effect of each mother’s attachment style on aggression. This research result proved that mother’s attachment continues to develop since childhood, which forms self-esteem and affects an adolescent’s aggressive behavior. This finding also suggested that mother’s secure attachment and high self-esteem in adolescents helped prevent aggression. This study supports the previous research stating that self-esteem mediated the relationship between mother’s attachment and aggressive behavior in adolescents [78,90].

Besides, the study explained that a positive parenting process (intimacy, monitoring, and peer approval) was related to the level of self-esteem in adolescents as well as the level of aggression [91].

This study contributed to the literature on preventing adolescent aggression, especially “*Klitih*” behavior in the special region of Yogyakarta. This study proves that mothers have an essential role in forming adolescent self-esteem and controlling aggressive behavior. This research can be used as literature to increase adolescent self-esteem through mother–child attachment. This study also reveals that several adolescents have low self-esteem and are judged to have insecure attachments and avoidant attachments. The relationships with primary educators are vital in determining children’s emotional growth. Besides, our study also contributed to parenting practice by paying more attention to how mothers raise their children. It is essential to educate parents about building secure mother–child attachment to allow children to have emotional well-being in the future.

The limitation of the study lies in the data collection method, in which case there was only one technique used. The results would be more accurate if various data collection methods were used. Considering that culture might influence aggression, the use of sample units distinguished based on culture may provide a more significant contribution. Therefore, future research can take advantage of cultural differences in each region in Indonesia and examine the different causes of each form of aggression (verbal and physical) to provide more prosperous and more detailed findings to prevent or overcome aggressive behavior in adolescents.

## 6. Conclusions

The present study is the first research in the Indonesian context that examined the role of self-esteem as a mediator for the effect of the mother’s attachment styles on aggression. The hypothesized model showed a good fit, indicating that self-esteem could enhance the effect of each mother’s attachment style on aggression. Our study showed that each mother’s attachment style (i.e., secure, insecure, and anxious attachment) significantly affected aggression. It was also found that each mother’s attachment style significantly affected self-esteem. In addition, the present study showed that self-esteem negatively affected aggression. These findings suggested that better mother’s attachment and higher self-esteem in adolescents could reduce the possibility of aggression whereas insecure attachment, anxious attachment, and low self-esteem would increase the risk of aggression.

## Figures and Tables

**Table 1:** Interpretation of correlation coefficient strength.

Correlation Coefficient	Interpretation
1	Perfect
0.70–0.99	Strong
0.40–0.699	Moderate
0.10–0.399	Weak
0	Zero

**Table 2:** Variables’ descriptive data.

Variable	Mean	Std. Deviation	Minimum	Maximum
Aggression	130.24	34.910	53.0	216.0
Secure attachment	71.91	21.548	18.0	126.0
Insecure attachment	32.45	9.867	8.0	56.0
Anxious attachment	20.03	6.272	5.0	35.0
Self-esteem	140.66	39.233	53.0	234.0
N = 730				

**Table 3:** Correlation between secure attachment, insecure attachment, anxious ambivalent attachment, self-esteem, and aggression.

Variable	Aggression	Self-Esteem
Secure attachment	−0.216 ***	0.421 ***
Insecure attachment	0.141 ***	−0.280 ***
Anxious attachment	0.172 ***	−0.340 ***
Self-esteem	−0.402 ***	

Note: *** *p* < 0.001.

**Table 4:** The results of hypothesis analysis.

Path	Coefficient (β)	*p*-Value
Direct effect		
Secure attachment -> self-esteem	0.402	0.000 ***
Insecure attachment -> self-esteem	−0.267	0.000 ***
Anxious attachment -> self-esteem	−0.314	0.000 ***
self-esteem -> aggression	−0.441	0.000 ***
Secure attachment -> aggression	−0.225	0.000 ***
Insecure attachment -> aggression	0.148	0.000 ***
Anxious attachment -> aggression	0.174	0.000 ***
Indirect effect		
Secure attachment -> self-esteem -> aggression	−0.177	0.000 ***
Insecure attachment -> self-esteem -> aggression	0.118	0.000 ***
Anxious attachment -> self-esteem -> aggression	0.138	0.000 ***
Total effect		
Secure attachment -> aggression	−0.402	0.000 ***
Insecure attachment -> aggression	0.265	0.000 ***
Anxious attachment -> aggression	0.312	0.000 ***

Note: *** *p* < 0.001.

## Data Availability

Not applicable.

## References

[1] Archer J., Coyne S.M. (2005). An Integrated Review of Indirect, Relational, and Social Aggression. Pers. Soc. Psychol. Rev..

[2] Marcus R.F. (2017). The Development of Aggression and Violence in Adolescence.

[3] Chandler A.B., Lawrence E. (2021). Covariations among attachment, attributions, self-esteem, and psychological aggression in early marriage. J. Fam. Psychol..

[4] Worth M.R., Smith S.W., Poling D.V. (2021). Students with Emotional and Behavioral Disorders and Verbal Aggression: Why School Professionals Should Care and What They Can Do. Beyond Behav..

[5] Sturmey P. (2017). The Wiley Handbook of Violence and Aggression.

[6] Schwartz D., Lansford J.E., Dodge K.A., Pettit G.S., Bates J.E. (2015). Peer Victimization During Middle Childhood as a Lead Indicator of Internalizing Problems and Diagnostic Outcomes in Late Adolescence. J. Clin. Child Adolesc. Psychol..

[7] Troop-Gordon W., Rudolph K.D., Sugimura N., Little T.D. (2015). Peer Victimization in Middle Childhood Impedes Adaptive Responses to Stress: A Pathway to Depressive Symptoms. J. Clin. Child Adolesc. Psychol..

[8] Mazzone A., Yanagida T., Caravita S.C.S., Strohmeier D. (2019). Moral Emotions and Moral Disengagement: Concurrent and Longitudinal Associations with Aggressive Behavior Among Early Adolescents. J. Early Adolesc..

[9] Kwak M., Oh I. (2017). Comparison of psychological and social characteristics among traditional, cyber, combined bullies, and non-involved. Sch. Psychol. Int..

[10] Cabello R., Gutiérrez-Cobo M.J., Fernández-Berrocal P. (2017). Parental Education and Aggressive Behavior in Children: A Moderated-Mediation Model for Inhibitory Control and Gender. Front. Psychol..

[11] Waller R., Hyde L.W., Klump K.L., Burt S.A. (2018). Parenting Is an Environmental Predictor of Callous-Unemotional Traits and Aggression: A Monozygotic Twin Differences Study. J. Am. Acad. Child Adolesc. Psychiatry.

[12] Estévez E., Jiménez T.I., Moreno D. (2018). Aggressive behavior in adolescence as a predictor of personal, family, and school adjustment problems. Psicothema.

[13] Najman J.M., Plotnikova M., Horwood J., Silins E., Fergusson D., Patton G.C., Olsson C., Hutchinson D., Degenhardt L., Tait R. (2019). Does adolescent heavier alcohol use predict young adult aggression and delinquency? Parallel analyses from four Australasian cohort studies. Aggress. Behav..

[14] Edwards P., Van De Mortel T., Stevens J. (2019). Perceptions of anger and aggression in rural adolescent Australian males. Int. J. Ment. Health Nurs..

[15] Sullivan T.N., Ross K.M., Carlson M.M., Hitti S.A., Behrhorst K.L. (2020). Implementation of Violence Prevention Programs. Handbook of Research on Emotional and Behavioral Disorders: Interdisciplinary Developmental Perspectives on Children and Youth.

[16] Paquin S., LaCourse E., Brendgen M., Vitaro F., Dionne G., Tremblay R.E., Boivin M. (2017). Heterogeneity in the development of proactive and reactive aggression in childhood: Common and specific genetic—Environmental factors. PLoS ONE.

[17] Poling D., Smith S.W., Taylor G.G., Worth M. (2019). Direct verbal aggression in school settings: A review of the literature. Aggress. Violent Behav..

[18] Taylor G.G., Smith S.W. (2019). Teacher Reports of Verbal Aggression in School Settings Among Students with Emotional and Behavioral Disorders. J. Emot. Behav. Disord..

[19] Potard C., Kubiszewski V., Fontaine R., Pochon R., Rusch E., Courtois R. (2014). Peer violence, mental health and suicidal ideation in a sample of French adolescent. Int. J. Ment. Health Promot..

[20] Kann L., McManus T., Harris W.A., Shanklin S.L., Flint K.H., Hawkins J., Queen B., Lowry R., Olsen E.O., Chyen D. (2016). Youth risk behavior surveillance—United States, 2015. Morb. Mortal. Wkly. Rep. Surveill. Summ..

[21] Rubio-Garay F., Carrasco M.A., Amor P.J. (2016). Aggression, anger and hostility: Evaluation of moral disengagement as a mediational process. Scand. J. Psychol..

[22] Rai T.S. (2019). Higher self-control predicts engagement in undesirable moralistic aggression. Pers. Individ. Differ..

[23] Elmasry N.M., Fouad A.A., Khalil D.M., Sherra K.S. (2016). Physical and verbal aggression among adolescent school students in Sharkia, Egypt: Prevalence and risk factors. Egypt. J. Psychiatry.

[24] Fitri S., Luawo M.I.R., Puspasari D. (2016). Gambaran Agresivitas Pada Remaja Laki-Laki Siswa SMA Negeri di DKI Jakarta. INSIGHT J. Bimbing. Konseling.

[25] Kasimova R.S., Biktagirova G.F. (2016). Art therapy as a means of overcoming aggressiveness in adolescents. Int. Electron. J. Math. Educ..

[26] Sharma D., Sangwan S. (2016). Impact of family environment on adolescents aggression. Adv. Res. J. Soc. Sci..

[27] Kumari P., Thapliyal S. (2017). Studying the impact of organizational citizenship behavior on organizational effectiveness. Hum. Resour. Manag..

[28] Naser A., Afshoon F., Mohammadi H.R., Tavasoli E., Fard M.R., Khalili A. (2017). Comparing the dimensions of aggression in adolescent athletes and non-athletes divorced families. Eur. J. Exp. Biol..

[29] Fasya H., Yasin S., Hafid A., Amelia A.F. (2017). Pengaruh Game Online Terhadap Tingkat Agresivitas Anak-anak dan Remaja di Kota Makassar (Studi Kasus di Kecamatan Tallo). Hasanuddin Stud. J..

[30] Sentana M.A., Kumala I.D. (2017). Agresivitas dan kontrol diri pada remaja di Banda Aceh. J. Sains Psikol..

[31] Afdal A., Fikri M., Pane N., Andriani W. (2020). Exploration of aggressive behavior among adolescent in Indonesia. Konselor.

[32] Erlin E. (2022). Miris, Sepanjang 2022 JPW Catat Ada 12 Kali Aksi Klitih di Yogyakarta. https://yogya.inews.id/berita/miris-sepanjang-2022-jpw-catat-ada-12-kali-aksi-klitih-di-yogyakarta/all.

[33] Sarmini M., Kurniyatuti N., Sukartiningsih S. Klithih: Invisible Crime by Teenagers. Proceedings of the 1st International Conference on Social Sciences (ICSS 2018).

[34] Karnadi A. (2022). Klitih Kembali Terjadi, Jumlah Kasusnya Naik 11,54% Pada 2021 Artikel ini Telah Tayang di Dataindone-sia.id Dengan Judul ‘Klitih Kembali Terjadi, Jumlah Kasusnya Naik 11,54% Pada 2021. https://dataindonesia.id/Ragam/detail/klitih-kembali-terjadi-jumlah-kasusnya-naik-1154-pada-2021.

[35] Priatmojo G. (2022). Polres Bantul Tangkap 104 Pelaku Kejahatan Jalanan Selama Periode Januari Hingga April 2022. https://jogja.suara.com/read/2022/04/18/211654/polres-bantul-tangkap-104-pelaku-kejahatan-jalanan-selama-periode-januari-hingga-april-2022.

[36] Casmini C., Supardi S. (2020). Family Resilience: Preventive Solution of Javanese Youth Klithih Behavior. Qual. Rep..

[37] Riany Y.E., Meredith P., Cuskelly M. (2017). Understanding the Influence of Traditional Cultural Values on Indonesian Parenting. Marriage Fam. Rev..

[38] Orth U., Erol R.Y., Luciano E.C. (2018). Development of self-esteem from age 4 to 94 years: A meta-analysis of longitudinal studies. Psychol. Bull..

[39] Brodie Z.P., Goodall K., Darling S., McVittie C. (2019). Attachment insecurity and dispositional aggression: The mediating role of maladaptive anger regulation. J. Soc. Pers. Relationships.

[40] Fagan A.A. (2020). Child maltreatment and aggressive behaviors in early adolescence: Evidence of moderation by parent/child relationship quality. Child Maltreat..

[41] McErlean A.B.J., Lim L.X.C. (2020). Relationship between Parenting Style, Alexithymia and Aggression in Emerging Adults. J. Fam. Issues.

[42] Granqvist P., Duschinsky R. (2021). Attachment Theory and Research.

[43] Strand P.S., Vossen J.J., Savage E. (2019). Culture and Child Attachment Patterns: A Behavioral Systems Synthesis. Perspect. Behav. Sci..

[44] Cricchio M.G.L., Musso P., Coco A.L., Cassibba R., Liga F. (2021). The Relation Between Empathy and Aggression: The Role of Attachment Style. Eur. J. Psychol..

[45] Cricchio M.G.L., Coco A.L., Cheah C.S.L., Liga F. (2019). The Good Parent: Southern Italian Mothers’ Conceptualization of Good Parenting and Parent–Child Relationships. J. Fam. Issues.

[46] Fraley R.C., Shaver P.R. (2021). Attachment Theory and its Place in Contemporary Personality Theory and Research. Handbook of Personality: Theory and Research.

[47] Sroufe L.A. (2021). Then and now: The legacy and future of attachment research. Attach. Hum. Dev..

[48] Bowlby J. (2008). A Secure Base: Parent-Child Attachment and Healthy Human Development.

[49] Nelson-Coffey S.K., Borelli J.L., River L.M. (2017). Attachment avoidance, but not anxiety, minimizes the joys of caregiving. Attach. Hum. Dev..

[50] Burkhart M.L., Borelli J.L., Rasmussen H.F., Brody R., Sbarra D.A. (2017). Parental mentalizing as an indirect link between attachment anxiety and parenting satisfaction. J. Fam. Psychol..

[51] Clear S.J., Zimmer-Gembeck M. (2017). Associations between Attachment and Emotion-Specific Emotion Regulation with and without Relationship Insecurity Priming. Int. J. Behav. Dev..

[52] Dubois-Comtois K., Moss E., Cyr C., Pascuzzo K. (2013). Behavior Problems in Middle Childhood: The Predictive Role of Maternal Distress, Child Attachment, and Mother-Child Interactions. J. Abnorm. Child Psychol..

[53] Guangdong Z. (2014). The Influence of Inter-Parental Conflict, Parenting Styles, and Attachment on Reactive and Proactive Aggression in Adolescence. Ph.D. Thesis.

[54] Kumar S.A., Mattanah J.F. (2016). Parental attachment, romantic competence, relationship satisfaction, and psychosocial adjustment in emerging adulthood. Pers. Relationships.

[55] Laible D.J., Thompson R.A. (2000). Attachment and Self-Organization. Emotion, Development, and Self-Organization.

[56] Rholes W.S., Paetzold R.L., Kohn J.L. (2016). Disorganized attachment mediates the link from early trauma to externalizing behavior in adult relationships. Pers. Individ. Differ..

[57] Curran T., Meter D., Janovec A., Brown E., Caban S. (2021). Maternal adult attachment styles and mother-child transmissions of social skills and self-esteem. J. Fam. Stud..

[58] Hart W., Richardson K., Breeden C.J. (2019). An interactive model of narcissism, self-esteem, and provocation extent on aggression. Pers. Individ. Differ..

[59] Queiroz P., Garcia O.F., Garcia F., Zacarés J.J., Camino C. (2020). Self and Nature: Parental Socialization, Self-Esteem, and Environmental Values in Spanish Adolescents. Int. J. Environ. Res. Public Health.

[60] Baumeister R.F., Campbell J.D., Krueger J.I., Vohs K.D. (2003). Does high self-esteem cause better performance, inter-personal success, happiness, or healthier lifestyles?. Psychol. Sci. Public Interes..

[61] Coopersmith S. (1967). The Antecedents of Self-Esteem.

[62] Hewitt J.P., Snyder C.R., Lopez S.J., Edwards L.M., Marques S.C. (2020). The social construction of self-esteem. The Oxford Handbook of Positive Psychology.

[63] D’Mello L., Monteiro M., Pinto N. (2018). A Study on the Self Esteem and Academic Performance among the Students. Int. J. Health Sci. Pharm..

[64] Harris M.A., Orth U. (2020). The link between self-esteem and social relationships: A meta-analysis of longitudinal studies. J. Pers. Soc. Psychol..

[65] Gomez T., Quiñones-Camacho L., Davis E. (2018). Building a Sense of Self: The Link between Emotion Regulation and Self-Esteem in Young Adults. UC Riverside Undergrad. Res. J..

[66] Donnellan M.B., Trzesniewski K.H., Robins R.W., Moffitt T.E., Caspi A. (2005). Low Self-Esteem Is Related to Aggression, Antisocial Behavior, and Delinquency. Psychol. Sci..

[67] Mikulincer M., Shaver P.R. (2005). Mental representations of attachment security: Theoretical foundation for positive social psychology. Interpersonal Cognition.

[68] Webster G.D., Kirkpatrick L.A., Nezlek J.B., Smith C., Paddock E.L. (2007). Different slopes for different folks: Self-esteem instability and gender as moderators of the relationship between self-esteem and attitudinal aggression. Self Identity.

[69] Garofalo C., Holden C.J., Zeigler-Hill V., Velotti P. (2016). Understanding the connection between self-esteem and aggression: The mediating role of emotion dysregulation. Aggress. Behav..

[70] Orth U., Robins R.W. (2019). Development of self-esteem across the lifespan. Handbook of Personality Development.

[71] Thomaes S., Stegge H., Olthof T. (2007). Externalizing shame responses in children: The role of fragile-positive self-esteem. Br. J. Dev. Psychol..

[72] Falkenbach D.M., Howe J.R., Falki M. (2013). Using self-esteem to disaggregate psychopathy, narcissism, and aggression. Pers. Individ. Differ..

[73] Barnett M.D., Powell H.A. (2016). Self-esteem mediates narcissism and aggression among women, but not men: A comparison of two theoretical models of narcissism among college students. Pers. Individ. Differ..

[74] Foster J.D., Kernis M.H., Goldman B.M. (2007). Linking adult attachment to self-esteem stability. Self Identity.

[75] Amad S., Gray N.S., Snowden R.J. (2021). Self-Esteem, Narcissism, and Aggression: Different Types of Self-Esteem Predict Different Types of Aggression. J. Interpers. Violence.

[76] Kalemi G., Michopoulos I., Efstathiou V., Tzeferakos G., Gkioka S., Gournellis R., Douzenis A. (2019). Self-esteem and aggression in women: Differences between female prisoners and women without criminal records. Women Health.

[77] Li S., Zhao F., Yu G. (2019). Ostracism and aggression among adolescents: Implicit theories of personality moderated the mediating effect of self-esteem. Child. Youth Serv. Rev..

[78] Gomez R., McLaren S. (2007). The inter-relations of mother and father attachment, self-esteem and aggression during late adolescence. Aggress. Behav..

[79] Baron R.A., Neuman J.H. (1996). Workplace violence and workplace aggression: Evidence on their relative frequency and potential causes. Aggress. Behav..

[80] Ainsworth M.S. (1979). Infant–mother attachment. Am. Psychol..

[81] Baumeister R.F., Heatherton T.F., Tice D.M. (1993). When ego threats lead to self-regulation failure: Negative consequences of high self-esteem. J. Pers. Soc. Psychol..

[82] Dutton K.A., Brown J.D. (1997). Global self-esteem and specific self-views as determinants of people’s reactions to success and failure. J. Pers. Soc. Psychol..

[83] Dancey C.P., Reidy J. (2007). Statistics without Maths for Psychology.

[84] Akoglu H. (2018). User’s guide to correlation coefficients. Turk. J. Emerg. Med..

[85] Hair J.F., Black W.C., Babin B.J., Anderson R.E., Tatham R.L. (2010). Multivariate Data Analysis: A Global Perspective.

[86] Laible D.J., Carlo G., Roesch S.C. (2004). Pathways to self-esteem in late adolescence: The role of parent and peer attachment, empathy, and social behaviours. J. Adolesc..

[87] Keizer R., Helmerhorst K.O.W., Gelderen L.V.R.-V. (2019). Perceived Quality of the Mother-Adolescent and Father-Adolescent Attachment Relationship and Adolescents’ Self-Esteem. J. Youth Adolesc..

[88] Rosen H.M. (2016). Seeking self-certainty in an uncertain time: Attachment style and self-esteem in emerging adulthood. Student Works.

[89] Clucas C. (2020). Understanding Self-Respect and Its Relationship to Self-Esteem. Pers. Soc. Psychol. Bull..

[90] Simons R.L., Simons L.G., Chen Y.-F., Brody G.H., Lin K.-H. (2007). Identifying the Psychological Factors That Mediate the Association Between Parenting Practices and Delinquency. Criminology.

[91] Özdemir Y., Vazsonyi A.T., Çok F. (2017). Parenting processes, self-esteem, and aggression: A mediation model. Eur. J. Dev. Psychol..

